# Supplementary material for: Rebuilding core abscisic acid signaling pathways of Arabidopsis in yeast
Source: EMBO J. 2019 Aug 1;38(17):e101859. doi: 10.15252/embj.2019101859 (PMC6717914; doi:10.15252/embj.2019101859)

SourceData Fig. 4B_Insert

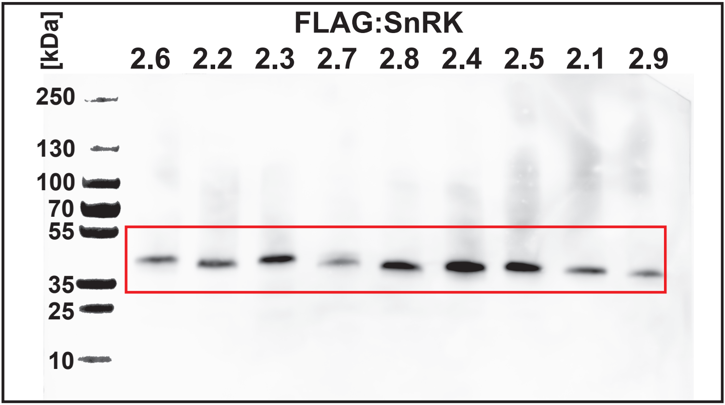

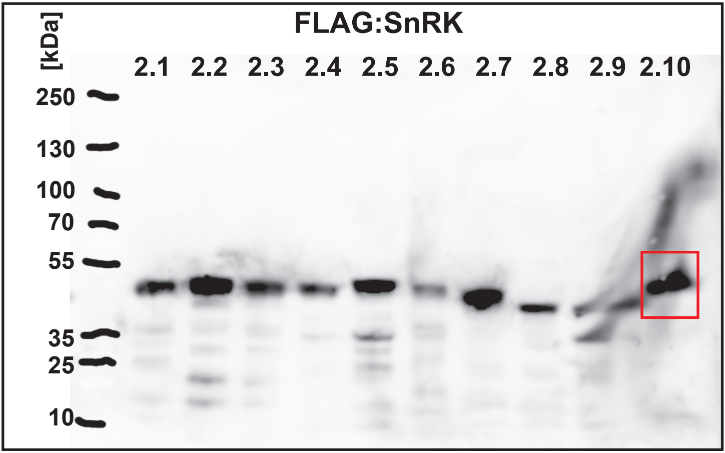

Supplement: Supplementary file 7 — Source Data for Figure 4 [file Figure_4_Source_Data.pdf › Figure_4_Source_Data.pdf]
