# Supplementary material for: Rebuilding core abscisic acid signaling pathways of Arabidopsis in yeast
Source: EMBO J. 2019 Aug 1;38(17):e101859. doi: 10.15252/embj.2019101859 (PMC6717914; doi:10.15252/embj.2019101859)
Supplement: Supplementary file 2 — Expanded View Figures PDF [file EMBJ-38-e101859-s002.pdf]

Expanded View Figures

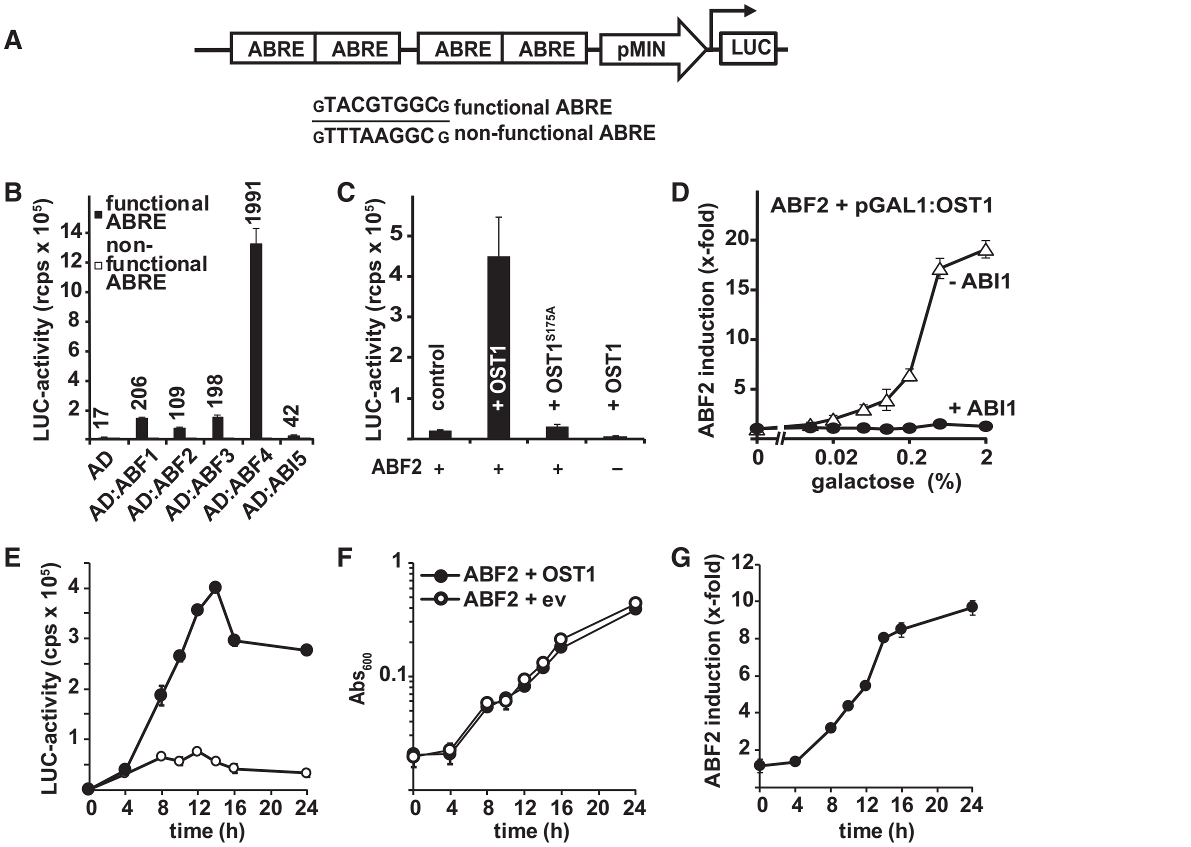

Figure EV1. Functional characterization of the ABA-responsive promoter in yeast.

A Schematic drawing of the reporter stably integrated into the *LYS2* locus of yeast. The ABA-responsive promoter contains four repeats of the ABRE (TACGTGGC) upstream of a minimal promoter (pMIN) driving the expression of the firefly luciferase gene (LUC). A promoter containing non-functional ABREs (TTTAAGGC) was used as negative control.

B Activation of the reporter gene by ABRE-specific binding of bZIP transcription factors ABF1–ABF4, and ABI5 fused to the activation domain of GAL4 (AD). The numbers indicate fold induction compared to the non-functional ABRE promoter.

C ABF2-mediated reporter activation by OST1. The mutant $OST1^{S175A}$ is impaired in auto-activation (Belin *et al*, 2006) and induced LUC expression twofold, while wild-type OST1 induced 29-fold.

D Regulation of ABF2-mediated reporter activity by differential induction of OST1 expression in the presence or absence of constitutively expressed ABI1. OST1 was expressed under the galactose-inducible promoter GAL1. OST1 induction was triggered by transfer of yeast cells into growth medium that contained different levels of galactose supplemented with raffinose to 2% sugar content. The LUC activity was determined 16 h after transfer in inducing medium. The LUC activity was normalized to the cell density and referred to samples expressing ABF2 alone.

E, F Time course of (E) ABF2-mediated induction of luciferase after cell transfer into 2% galactose-containing medium and (F) growth assessed by absorbance at 600 nm of cells coexpressing OST1 or empty vector (ev).

G ABF2 induction by OST1 based on the data shown in (E, F). The luciferase activity (E) was normalized by the cell density (F) and expressed relative to the control sample expressing only ABF2.

Data information: The data represent the mean ± SD; *n* = 6 biological replicates derived from three independent yeast transformants for (B, C); *n* = 3 biological replicates derived from one yeast line for (D–G).

© 2019 The Authors

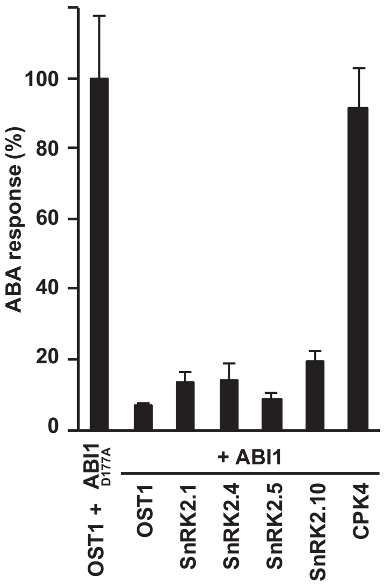

Figure EV2. Inhibition of ABF2-induced reporter expression by the ABA coreceptor ABI1.

SnRK2- and ABF2-mediated reporter expression was inhibited by ABI1 but not by the catalytically inactive ABI1^{D177A}. The ABA response is presented in relation to controls expressing ABI1^{D177A}. Reporter activity of control samples was 0.13×10^5, 1.4×10^5, 1.2×10^5, 1.0×10^5, 0.15×10^5, and 1.1×10^5 rcps for OST1, SnRK2.1, SnRK2.4 SnRK2.5, SnRK2.10, and CPK4, respectively.
Data information: The data represent the mean ± SD; $n = 9$ biological replicates derived from three independent yeast transformants.

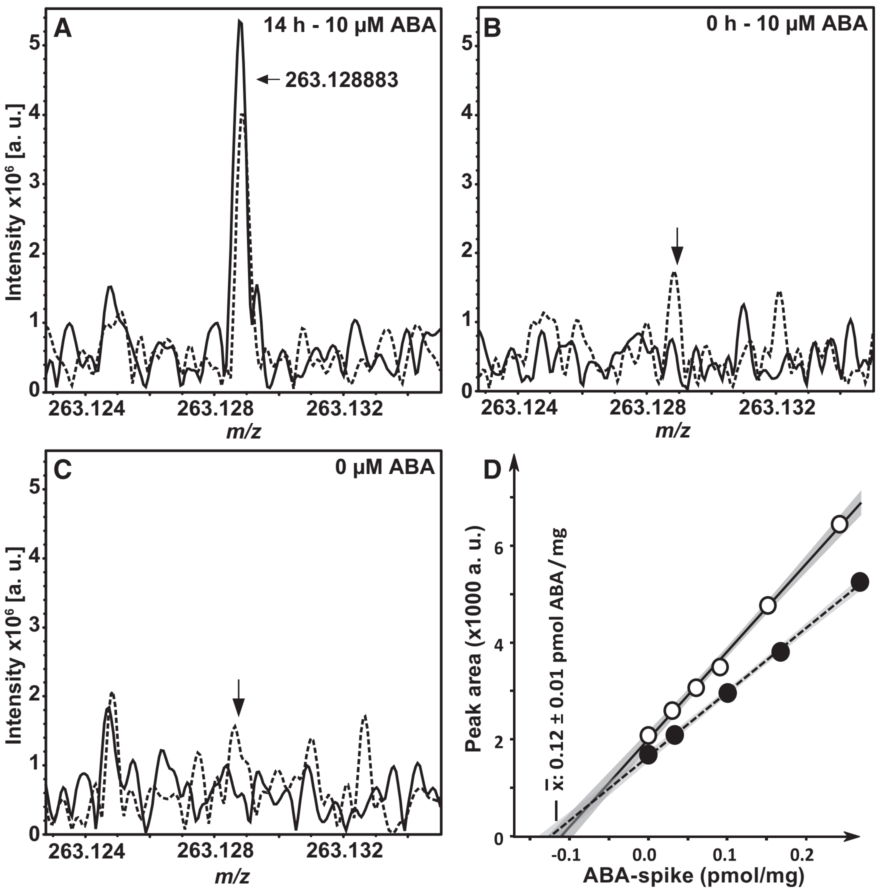

Figure EV3. Analysis of ABA uptake into yeast by Fourier-transform ion cyclotron resonance mass spectrometry.

A–C Spectral data for *m/z* values close or identical to the ABA signal (±6 mDa). Methanol extracts of yeast cells exposed to 10 µM ABA for 14 h (A) or 0 h (B), or not exposed to the phytohormone (C) were analyzed for the presence of ABA ($n = 2$, solid and interrupted lines). Arrows in (A–C) indicate the position of the exact mass of ABA $m/z = 263.128883$. The average measured *m/z* value of the recorded signals in (A) was 263.128857 (error = −0.1 ppm).

D Quantification of ABA uptake. The methanolic extracts from (A) were spiked with ABA prior to mass spectrometry, and the ABA-specific peak area was plotted relative to the picomole amount of ABA added per mg yeast material (density 1.1 g/ml) in two independent experiments (filled and open circles), respectively. Linear regression ($r > 0.999$) allowed calculating the mean ABA content per mg yeast fresh weight. Gray shaded areas indicate the 95% confidence intervals.

 © 2019 The Authors

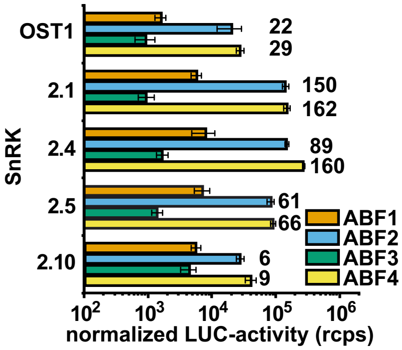

Figure EV4. ABF-mediated reporter activity induced by SnRK2 members normalized to the transactivation of the respective ABFs fused to the GAL4 activation domain.

The data of ABF-mediated reporter induction by SnRK2s as shown in Fig 4C were normalized to the activation of ABF fusion proteins containing the GAL4 activation domain as shown in Fig EV1B in the absence of SnRK2. The induction level with ABF2 and ABF4 in comparison with the normalized values for ABF3 is given on top of the column.

Data information: The data represent the mean ± SD; $n = 9$ biological replicates derived from three independent yeast transformants.

© 2019 The Authors

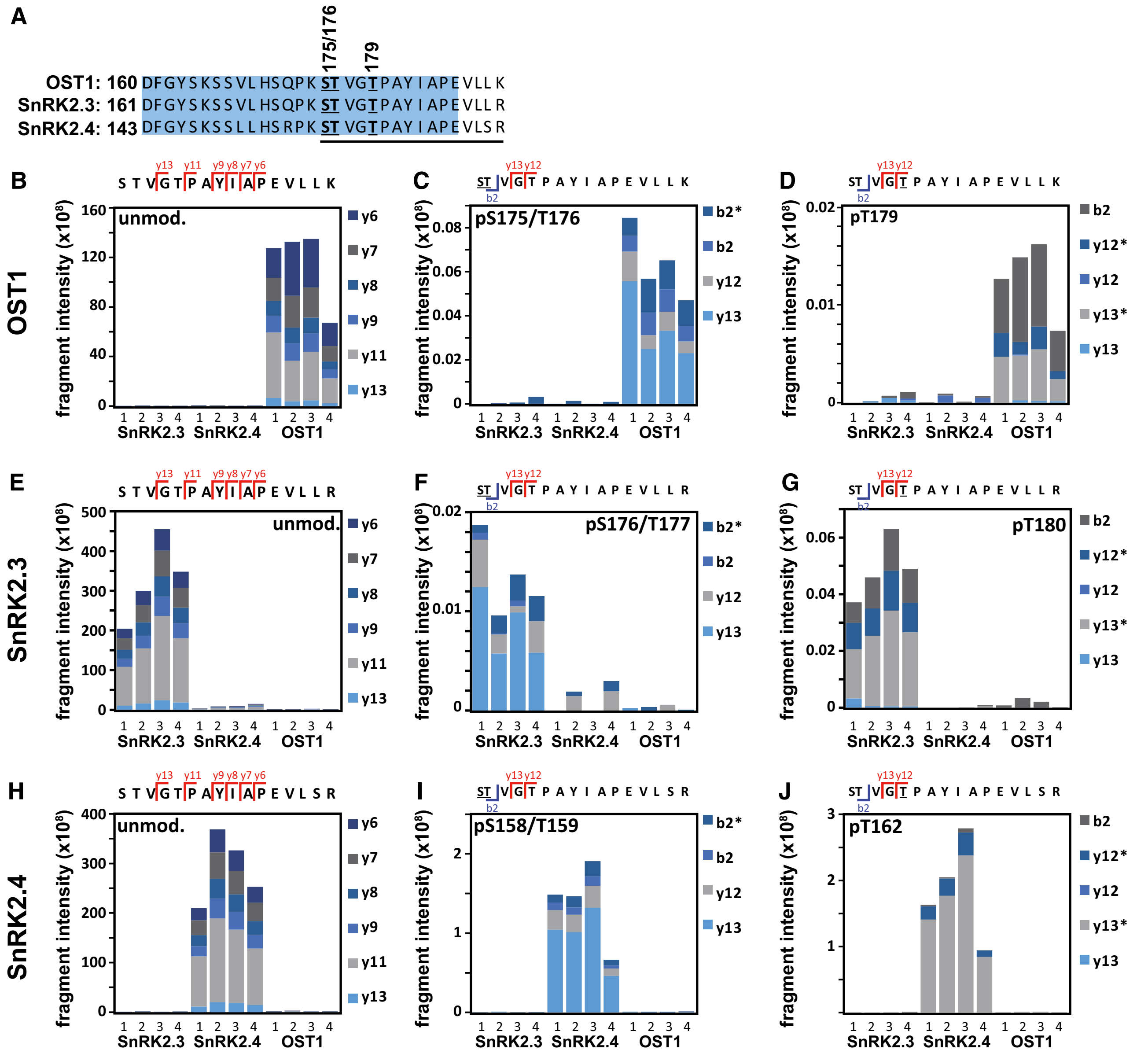

Figure EV5. Quantification of SnRK2 phosphorylation sites within the activation loop.

A Peptide sequences of OST1, SnRK2.3, and SnRK2.4 covering the activation loop highlighted in blue color. The underlined sequence corresponds to the analyzed peptide, and phosphorylated residues are underlined.

B–J Quantitative readout of parallel reaction monitoring assay transitions for selected SnRK2 phospho-peptides and their respective unmodified counterparts. (B–D) Quantification of OST1, (E–G) SnRK2.3, and (H–J) SnRK2.4 for the unmodified peptide (B, E, H), pS175/T176-phosphorylation and the corresponding sites for SnRK2.3 (pS176/T177), and SnRK2.4 (pS158/T159), (C, F, I), and pT179-phosphorylation and the corresponding sites of SnRK2.3 (pT180) and SnRK2.4 (pT162), (D, G, J). The site-determining ions of y-ion series (red) and b-ion series (blue) are shown for each peptide, and asterisks indicate phosphate group-neutral loss. Peptide fragments used for quantification are shown above each panel and are based on site-determining ions (Appendix Fig S3). Underlined residues indicate the phosphorylated form.

Data information: The data show the quantification of four independent replicates for each SnRK2.

 © 2019 The Authors
