# Supplementary material for: Rebuilding core abscisic acid signaling pathways of Arabidopsis in yeast
Source: EMBO J. 2019 Aug 1;38(17):e101859. doi: 10.15252/embj.2019101859 (PMC6717914; doi:10.15252/embj.2019101859)
Supplement: Supplementary file 1 — Appendix [file EMBJ-38-e101859-s001.pdf]

APPENDIX

Content

Appendix Table S1: DNA sequence of ABA-responsive reporter construct.
Appendix Table S2: Significance of ABA-independent and ABA-dependent RCAR effects.
Appendix Fig. S1: Effect of different promoters driving expression of ABI1 and RCAR11 on reporter regulation in yeast.
Appendix Fig. S2: Transactivation by SnRK2 members with deleted ABA-box and inhibition of OST1 variants by ABI1.

Appendix Fig. S3: Analysis of phosphorylation sites within peptides from the activation loop of OST1, SnRK2.3, and SnRK2.4.

Appendix Fig. S4: Comparable protein expression levels of OST1 and SnRK2.4 in yeast.

Appendix Table S1: DNA sequence of ABA-responsive reporter construct
The ABA-responsive reporter construct used in yeast experiments consists of four ABRE *cis*-elements (bold) positioned upstream of a 35S-minimal promotor (underlined), which is followed by the luciferase gene (red) and the NOS-terminator (blue). The restriction sites NotI and SalI (italic) were used for cloning the fragment into the yeast *LYS2* disintegration vector pIS385 (Sadowski et al, 2007).

*CGGCCG*CTCTAGCTAGCTAGCTAGCG**TACGTGGC**GG**TACGTGGC**GACTACTAGCG**TACGTGGC**GG**TACGTGGC**GA
CTACTAGTGCAAGACCCTTCCTCTATATAAGGAAGTTCATTTCATTTGGAGAGAACACGGGGGACTCTAGTGGAT
CCGAGCTTGGAATTCCTTTGTGTTACATTCTTGAATGTCGCTCGCAGTGACATTAGCATTCCGGTACTGTTGGTA
AAATGGAAGACGCCAAAAACATAAAGAAAGGCCCGGCGCCATTCTATCCTCTAGAGGATGGAACCGCTGGAGAGC
AACTGCATAAGGCTATGAAGAGATACGCCCTGGTTCCTGGAACAATTGCTTTTACAGATGCACATATCGAGGTGA
ACATCACGTACGCGGAATACTTCGAAATGTCCGTTCGGTTGGCAGAAGCTATGAAACGATATGGGCTGAATACAA
ATCACAGAATCGTCGTATGCAGTGAAAACTCTCTTCAATTCTTTATGCCGGTGTTGGGCGCGTTATTTATCGGAG
TTGCAGTTGCGCCCGCGAACGACATTTATAATGAACGTGAATTGCTCAACAGTATGAACATTTCGCAGCCTACCG
TAGTGTTTGTTTCCAAAAAGGGGTTGCAAAAAATTTTGAACGTGCAAAAAAAATTACCAATAATCCAGAAAATTA
TTATCATGGATTCTAAAACGGATTACCAGGGATTTCAGTCGATGTACACGTTCGTCACATCTCATCTACCTCCCG
GTTTTAATGAATACGATTTTGTACCAGAGTCCTTTGATCGTGACAAAACAATTGCACTGATAATGAATTCCTCTG
GATCTACTGGGTTACCTAAGGGTGTGGCCCTTCCGCATAGAACTGCCTGCGTCAGATTCTCGCATGCCAGAGATC
CTATTTTTGGCAATCAAATCATTCCGGATACTGCGATTTTAAGTGTTGTTCCATTCCATCACGGTTTTGGAATGT
TTACTACACTCGGATATTTGATATGTGGATTTCGAGTCGTCTTAATGTATAGATTTGAAGAAGAGCTGTTTTTAC
GATCCCTTCAGGATTACAAAATTCAAAGTGCGTTGCTAGTACCAACCCTATTTTCATTCTTCGCCAAAAGCACTC
TGATTGACAAATACGATTTATCTAATTTACACGAAATTGCTTCTGGGGGCGCACCTCTTTCGAAAGAAGTCGGGG
AAGCGGTTGCAAAACGCTTCCATCTTCCAGGGATACGACAAGGATATGGGCTCACTGAGACTACATCAGCTATTC
TGATTACACCCGAGGGGGATGATAAACCGGGCGCGGTCGGTAAAGTTGTTCCATTTTTTGAAGCGAAGGTTGTGG
ATCTGGATACCGGGAAAACGCTGGGCGTTAATCAGAGAGGCGAATTATGTGTCAGAGGACCTATGATTATGTCCG
GTTATGTAAACAATCCGGAAGCGACCAACGCCTTGATTGACAAGGATGGATGGCTACATTCTGGAGACATAGCTT
ACTGGGACGAAGACGAACACTTCTTCATAGTTGACCGCTTGAAGTCTTTAATTAAATACAAAGGATATCAGGTGG
CCCCCGCTGAATTGGAATCGATATTGTTACAACACCCCAACATCTTCGACGCGGGCGTGGCAGGTCTTCCCGACG
ATGACGCCGGTGAACTTCCCGCCGCCGTTGTTGTTTTGGAGCACGGAAAGACGATGACGGAAAAAGAGATCGTGG
ATTACGTCGCCAGTCAAGTAACAACCGCGAAAAAGTTGCGCGGAGGAGTTGTGTTTGTGGACGAAGTACCGAAAG
GTCTTACCGGAAAACTCGACGCAAGAAAAATCAGAGAGATCCTCATAAAGGCCAAGAAGGGCGGAAAGTCCAAAT
TGTAAAATGTAACTGTATTCAGCGATGACGAAATTCTTAGCTATTGTAATATTATATGCAAATTGATGAATGGTA
ATTTTGTAATTGTGGGTCACTGTACTATTTTAACGAATAATAAAATCAGGTATAGGTAACTAAAAAGGAATTCGA
GCTCGAATTTCCCCGATCGTTCAAACATTTGGCAATAAAGTTTCTTAAGATTGAATCCTGTTGCCGGTCTTGCGA
TGATTATCATATAATTTCTGTTGAATTACGTTAAGCATGTAATAATTAACATGTAATGCATGACGTTATTTATGA
GATGGGTTTTTATGATTAGAGTCCCGCAATTATACATTTAATACGCGATAGAAAACAAAATATAGCGCGCAAACT
AGGATAAATTATGCGCGCGGTGTCATCTATGTTACTAGATCGGGAATTCGATATCAAGCTTATCGATACC*GTCGA*
C

Appendix Table S2: Significance of ABA-independent and ABA-dependent RCAR effects.

Statistical analysis to Fig. 2A.

ABA-independent RCAR induction<br>Welch-corrected one sided t-test; α=0.05			ABA-dependent RCAR induction<br>Welch-corrected one sided t-test; α=0.05	
**control/no-ABA to RCAR/no-ABA**	**p-value**		**RCAR/no ABA to RCAR/ 0.1 µM ABA**	**p-value**
RCAR1	4.43E-05		RCAR1	2.58E-11
RCAR2	9.99E-01		RCAR2	2.58E-11
RCAR3	1.05E-08		RCAR3	1.47E-06
RCAR4	1.37E-08		RCAR4	7.51E-01
RCAR5	9.80E-01		RCAR5	3.23E-13
RCAR6	3.31E-01		RCAR6	4.34E-07
RCAR7	9.63E-01		RCAR7	3.81E-01
RCAR8	1.00E+00		RCAR8	1.63E-08
RCAR9	7.32E-01		RCAR9	1.41E-08
RCAR10	1.00E+00		RCAR10	1.62E-08
RCAR11	1.00E+00		RCAR11	1.43E-08
RCAR12	1.00E+00		RCAR12	4.46E-08
RCAR13	7.12E-01		RCAR13	7.79E-11
RCAR14	1.00E+00		RCAR14	1.34E-09

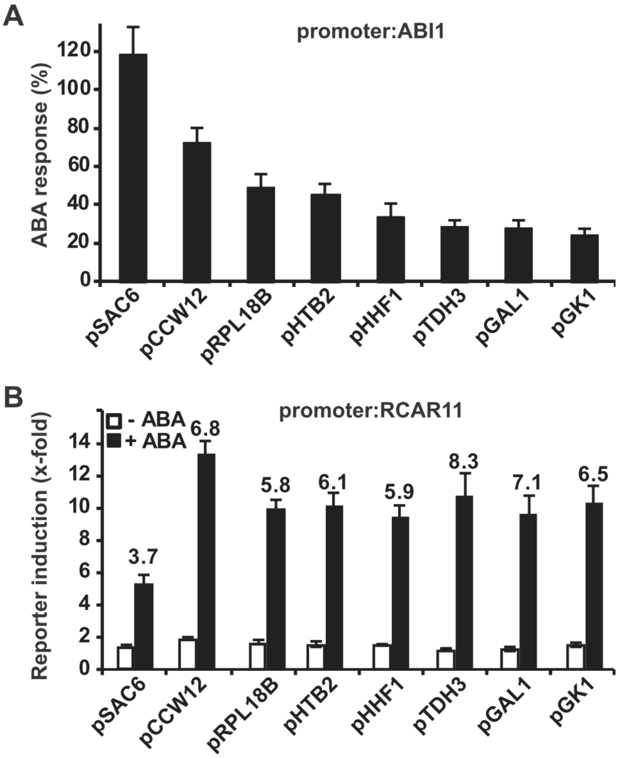

Appendix Fig. S1: Effect of different promoters driving expression of ABI1 and RCAR11 on reporter regulation.

A) The inhibition of ABF2- and OST1$^{\Delta320}$-mediated luciferase reporter activity was assayed by expression of ABI1 under the control of different yeast promoters as indicated.

B) Regulation of the ABF2-OST1$^{\Delta320}$-ABI1 pathway by expression of RCAR11 in the absence or presence of 0.1 mM ABA. ABI1 was expressed under the control of the constitutive active GK1-promoter and a variety of promoters were used as indicated for RCAR11 expression. The numbers on top of the columns indicate the fold induction by ABA. Based on these results, the GK1- and TDH3-promotor were used in subsequent analyses for expression of PP2Cs and RCARs, respectively.

Data information: The data represent the mean ± s.d; n = 6 biological replicates derived from three independent yeast transformants. In (A) yeast cells expressing no ABI1 served as 100 % control while in (B) the activity of samples without RCAR 11 was set to 1 for both treatments.

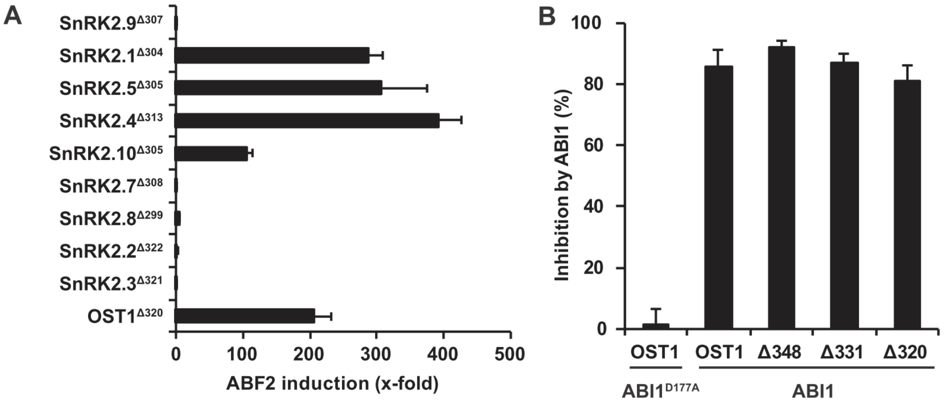

Appendix Fig. S2: Transactivation by SnRK2 members with deleted ABA-box and inhibition of OST1 variants by ABI1.

A) The ABA-box of SnRK2s was removed and the truncated protein kinases examined for transactivation of ABF2 in yeast.

B) Efficiency of ABI1 to inhibit the transactivation of OST1 and versions with partial (Δ348, Δ331) or complete (Δ320) deletion of the ABA-box. Samples without ABI1 expression were set to 100% activity and the catalytically inactive ABI1^{D177A} served as additional control.

Data information: The data represent the mean ± s.d; n = 6 biological replicates derived from three independent yeast transformants.

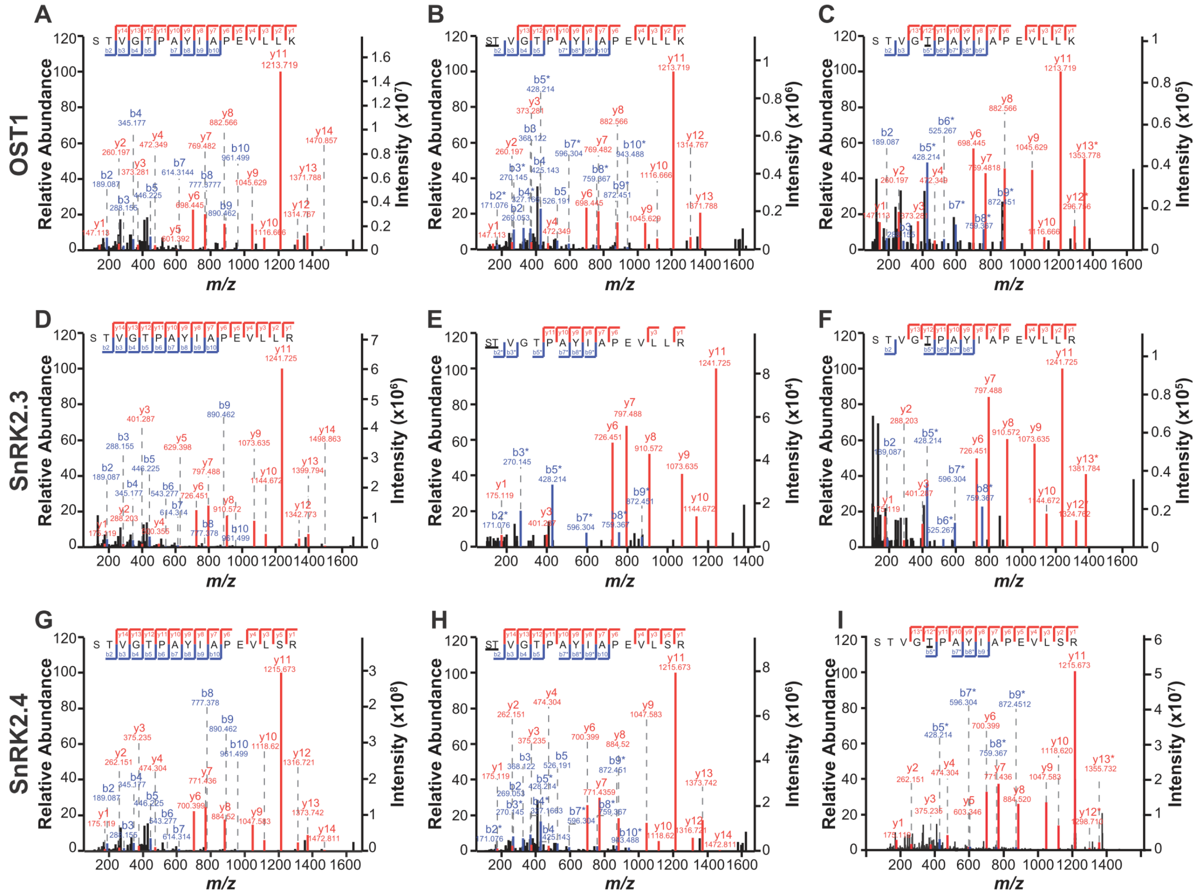

Appendix Fig. S3. Analysis of phosphorylation sites within peptides from the activation loop of OST1, SnRK2.3, and SnRK2.4.

Representative annotated ms/ms spectra for selected OST1 phosphorylation sites (A, B, C) and corresponding sites in SnRK2.3 (D, E, F) and SnRK2.4 (G, H, I). Phosphorylated residues are underlined in the corresponding peptide sequence (on top of each spectrum). Fragment ions of y-ion series (red) and b-ion series (blue) are shown for each peptide and asterisks indicate phosphate group-neutral loss. (A, D, G) Spectra for unmodified peptides, (B) for OST1-pS175/T176, (E) SnRK2.3-p176/T177, (H) SnRK2.4-pS158/T159, (C) OST1-pT179, (F) SnRK2.3-p180, and (I) SnRK2.4-pT162. One spectrum (I) could not be obtained and was extracted from parallel reaction monitoring experiments.

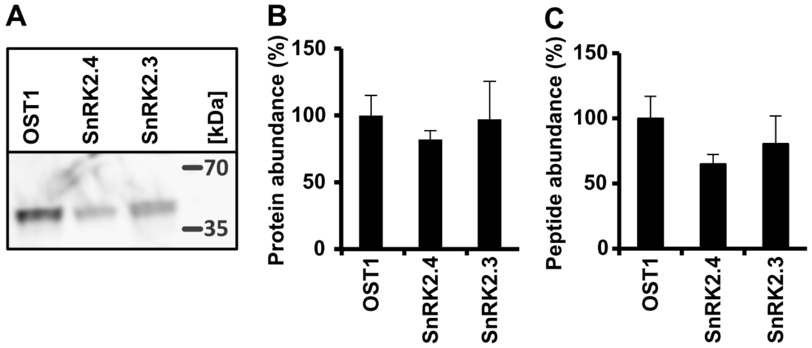

Appendix Fig. S4: Comparable protein expression levels of OST1 and SnRK2.4 in yeast.

A) OST1, SnRK2.2, and SnRK2.3 were immuno-detected by Western blotting of 10 µg yeast extracts by FLAG-specific antibodies.

B) Quantitative dot-blot analysis of 100 ng yeast extracts with the FLAG immuno-signal for OST1 set to 100%.

C). Relative peptide intensity of the SnRK2-peptides identified by mass spectrometric analysis normalized to the overall peptide intensity. Signal for OST1 is set to 100%.

Data Information: The data in (B) and (C) represent the mean ± s.d. of four biological replicates**.**
